# A case report of pediatric acute lymphoblastic leukemia with e8a2 *BCR/ABL1* fusion transcript

**DOI:** 10.1186/s12920-022-01169-0

**Published:** 2022-02-05

**Authors:** Aleksandra Mroczkowska, Bożena Jaźwiec, Justyna Urbańska-Rakus, Sylwia Szymanowska, Anna Tessmann, Sonia Pająk, Katarzyna Machnik, Olga Haus, Tomasz Wróbel

**Affiliations:** 1 Laboratory of Molecular Biology and Cytogenetics, Department of Hematology, Blood Neoplasms and Bone Marrow Transplantation, Wroclaw Medical University, Wrocław, Poland; 2 Department of Hematology, Blood Neoplasms and Bone Marrow Transplantation, Wroclaw Medical University, Wrocław, Poland; 3 Unit of Pediatric Hematology and Oncology, City Hospital, Chorzow, Poland; 4 Department of Clinical Genetics, Faculty of Medicine, Collegium Medicum in Bydgoszcz, Nicolaus Copernicus University in Torun, Bydgoszcz, Poland

**Keywords:** Acute lymphoblastic leukemia, *BCR/ABL1*, e8a2, RT-PCR, FISH, Case report

## Abstract

**Background:**

Acute lymphoblastic leukemia is the most common type of cancer in children. Most often it affects the age group between 2 and 5 years of age. Studies have shown an improvement in general survivability, more than 90% 5-year overall survival (OS). Current treatment protocols for acute lymphoblastic leukemia require verification of the presence of favorable and unfavorable genetic abnormalities, which help qualify patients to the appropriate risk group and select a more suitable treatment. The presence of the *BCR/ABL1* fusion gene stratifies the patient into a high-risk group and requires special treatment with tyrosine kinase inhibitors (TKI). The three dominant mRNA transcripts are e1a2, e13a2, and e14a2. Nevertheless, cases of atypical *BCR/ABL1* transcripts have also been reported.

**Case presentation:**

This paper presents the case of a pediatric patient with Ph + B-cell precursor acute lymphoblastic leukemia with rare atypical e8a2 *BCR/ABL1* fusion transcript. Our patient achieved complete remission after 33 days of treatment. Molecular and cytogenetic studies in TP1 did not reveal the presence of the *BCR/ABL1* transcript. The PCR-MRD test in TP1b was negative, the patient did not require hematopoietic stem cell transplantation.

**Conclusion:**

Genetic evaluation of the bone marrow sample is crucial in the initial stage of the diagnosis. Fluorescent in situ hybridization and reverse transcriptase polymerase chain reaction with Sanger sequencing are the appropriate methods used in the detection of rare variants of *BCR/ABL1* transcripts.

## Background

Acute lymphoblastic leukemia (ALL) is the most common childhood malignancy. ALL is a heterogeneous neoplasm derived from the precursors of the lymphoid lineage. About 80–85% of cases are B-cell precursor leukemias, while T-lineage leukemias are about 15–20%. The ALL diagnoses are based on certain criteria including clinical presentation, laboratory tests, a bone marrow biopsy, immunophenotypic analysis and genetic tests. Currently, cytogenetic and molecular tests play a very important role in determining prognosis and stratification for suitable treatment of pediatric ALL [1, 2]. The typical recurrent translocations occurring in ALL are t(12;21)(p13;q22) causing *ETV6/RUNX1*, t(1;19)(q23;p13) causing *TCF3/PBX1*, t(9;22)(q34;q11.2) causing *BCR/ABL1*, and the most common rearrangement of *KMT2A* gene, t(4;11)(q21;q23) causing *KMT2A/AFF1*. *ETV6/RUNX1* is associated with a favorable prognosis and the last three genetic abnormalities have unfavorable outcomes [3, 4].

*BCR/ABL1* fusion transcripts occur approximately in 2–5% cases of childhood ALL and the frequency of BCR/ABL1(+)ALL increases with the patient’s age [5]. ALL cases with this genetic abnormality are associated with poor outcome and are qualified to the high risk group. Due to the introduction of tyrosine kinase inhibitors to the therapy, the prognosis of Ph + patients has improved.

The most common mRNA transcripts of *BCR/ABL1:* e1a2, e13a2, e14a2, occur in about 99% of Ph + cases. Approximately 70% of Ph + ALL patients have an e1a2 transcript and more than 25% e13a2 or e14a2. 1% of patients with Ph + shows atypical transcripts like e19a2, e6a2, e1a3, e13a3, e14a3 and e8a2 [6].

We present here a case of a pediatric patient with Ph + BCP-ALL (B cell precursor ALL) with an e8a2 *BCR/ABL1* transcript.

## Case presentation

An 11-year-old boy was admitted to the Unit of Pediatric Hematology and Oncology, City Hospital, Chorzów, Poland due to a suspicion of acute leukemia. Five days before admission to the hospital, he developed a severe and difficult to stop nosebleed. Since then, the boy was experienced weakness, lethargy, lack of appetite. Additionally he developed abdominal pain, a headache and nausea. Physical examination revealed pale skin with petechiae, inflammation of the gingiva, tooth decay and splenomegaly. Lymphadenopathy, hepatomegaly and the presence of a Central Nervous System (CNS) disease/leukemia were not observed. Family Health History has no indication of any genetic, hematologic or cancerous diseases. Patient was not exposed to any physical (i.e. ionic radiation) or chemical factors (organic solvents, pesticides, herbicides, paints, lacquers) during childhood nor fetal period. He was born out of second pregnancy, first childbirth (first pregnancy ended due to spontaneous miscarriage around eighth week). Weight at birth 2400 g. Mother's age at birth: 19, father: 21. Patient has younger step-sister (same mother, different father), showing no symptoms of ALL or any other hematological disorders.

By the time of diagnosis of ALL, the patient had been sick sporadically and had no routine blood tests—including morphology. The patient has not taken any medications on a permanent basis.

The laboratory results showed: white blood cell 206,900/µl, platelet count 142,000/µl and hemoglobin level 10.2 g/dl. The bone marrow was highly cellular, represented by a homogeneous population of small blasts with lymphoid morphology (88.5%). Flow cytometric analysis showed BCP-ALL phenotype: CD45dim + , CD38 + , CD34(+), CD81(+), CD24(+), CD19(+), CD79a(+), TdT(+), CD10(+), CDdim33(+), CD20dim(+), CD22dim(+), CD15(-), CD117(-). The boy was diagnosed with common B-cell precursor ALL and qualified for treatment according to the AIEOP-BFM ALL 2017 protocol.

**Fig. 1:**
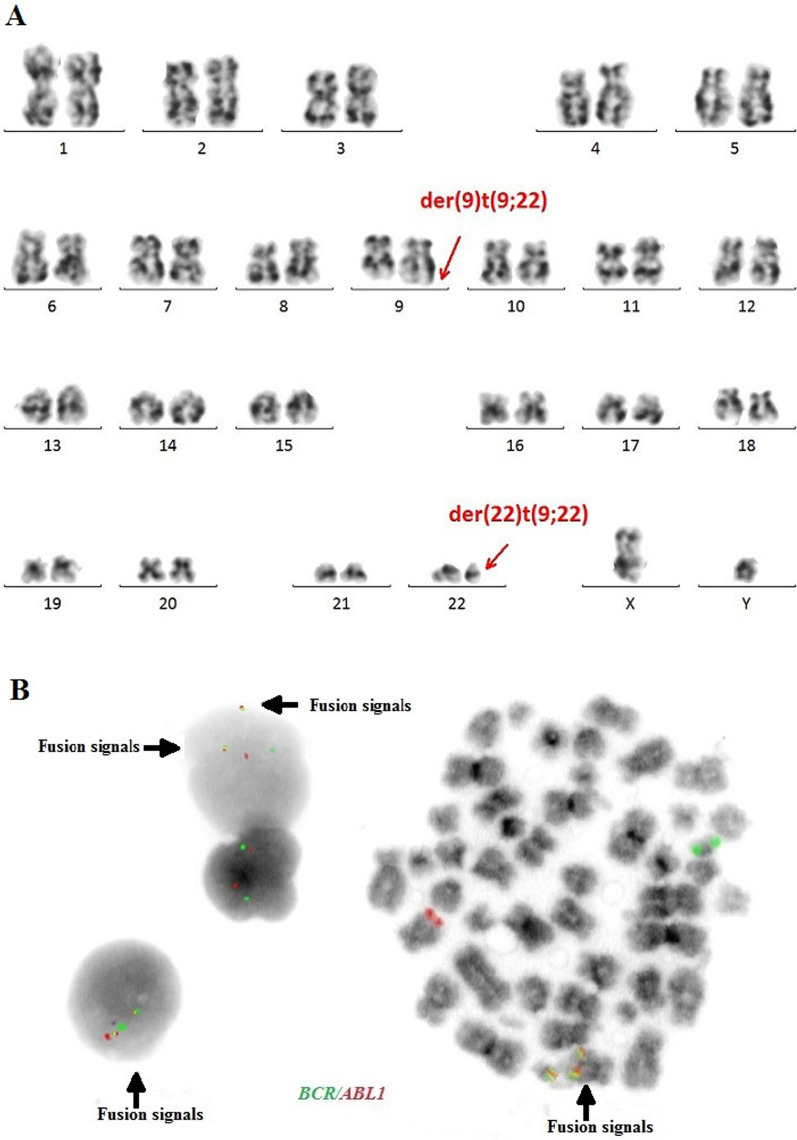
**A**—Conventional G-banding karyotype analysis showing typical translocation between chromosome 9 and 22. **B**—FISH analysis on interphase and metaphase with LSI BCR/ABL1 Dual Color, Dual Fusion Translocation Probe

**Fig. 2:**
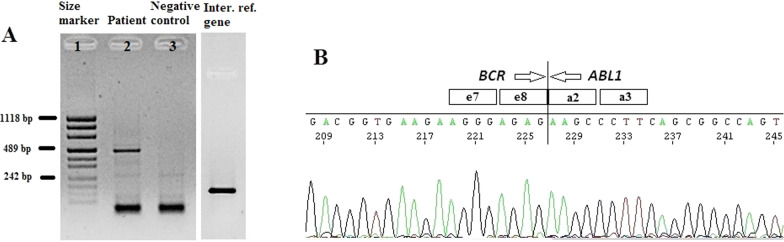
**A**—Detection of e8a2 BCR/ABL1 transcript by RT-PCR. Lane 1: size marker; lane 2: patient sample; lane 3: negative control, lane 4: internal reference gene—ABL1. **B**—Sanger sequencing demonstrating the direct junction between BCR exon e8 and ABL1 exon a2

The cytogenetic and molecular examinations of the patient's bone marrow were performed by the Laboratory of Molecular Biology and Cytogenetics at the University Clinical Hospital in Wroclaw. Karyotype analysis and fluorescence in situ hybridization (FISH) was performed on the bone marrow sample according to the AIEOP-BFM ALL 2017 protocol. According to the protocol, tests for genetic diagnostics were performed. By day 6, a FISH test was performed to obtain a result for the presence of the Philadelphia chromosome. Up to day 33, the FISH test was performed for the frequent genetic aberrations: ETV6/RUNX1 translocation and rearrangements in the KMT2A and TCF3 genes. At the same time, molecular tests were carried out using the RT-PCR method for the presence of the BCR/ABL1 and KMT2A/AFF1 fusion gene. G-banded chromosome analysis revealed an abnormal male karyotype 46,XY,t(9;22)(q34;q11) [11]/46,XY [9] (Fig. 1A). The FISH study showed no rearrangements in *ETV6/RUNX1, TCF3* (MetaSystems Probes, Germany) or *KMT2A* (Vysis, Abbott Molecular, Illinois, USA). The FISH study performed with the *BCR/ABL1* dual color, dual fusion translocation probe (Vysis, Abbott Molecular, Illinois, USA) disclosed a typical translocation pattern 2 green/orange *BCR/ABL1* fusion signals, 1 green BCR signal, and one orange *ABL1* signal in 90% of the interphase cells (Fig. 1B). Reverse transcription-polymerase chain reaction (RT-PCR) was performed to detect the presence or absence of the *KMT2A /AFF1* and *BCR/ABL1* fusion gene using primers as per JJM van Dongen et al. [7]. The test was negative in both cases. Due to the positive result of the FISH test for *BCR/ABL1*, another RT-PCR was performed in order to search for atypical *BCR/ABL1* transcripts. New RT-PCR analysis was performed based on primers BCR-6 and ABL-3 published by T. Burmeister and R. Reinhardt [6]. Electrophoresis showed a band of ~ 489 bp (Fig. 2A). Sanger sequencing confirmed the direct junction between exon 8 of *BCR* (NM_004327.4) and exon 2 of *ABL1* (NM_005157.6) (Fig. 2B). The Sanger sequencing was important because this method determined the type of transcript by analyzing the direct junction between exons. Transcript type information is crucial for monitoring the presence of BCR/ABL1 transcript by RT-PCR method.

**Fig. 3:**
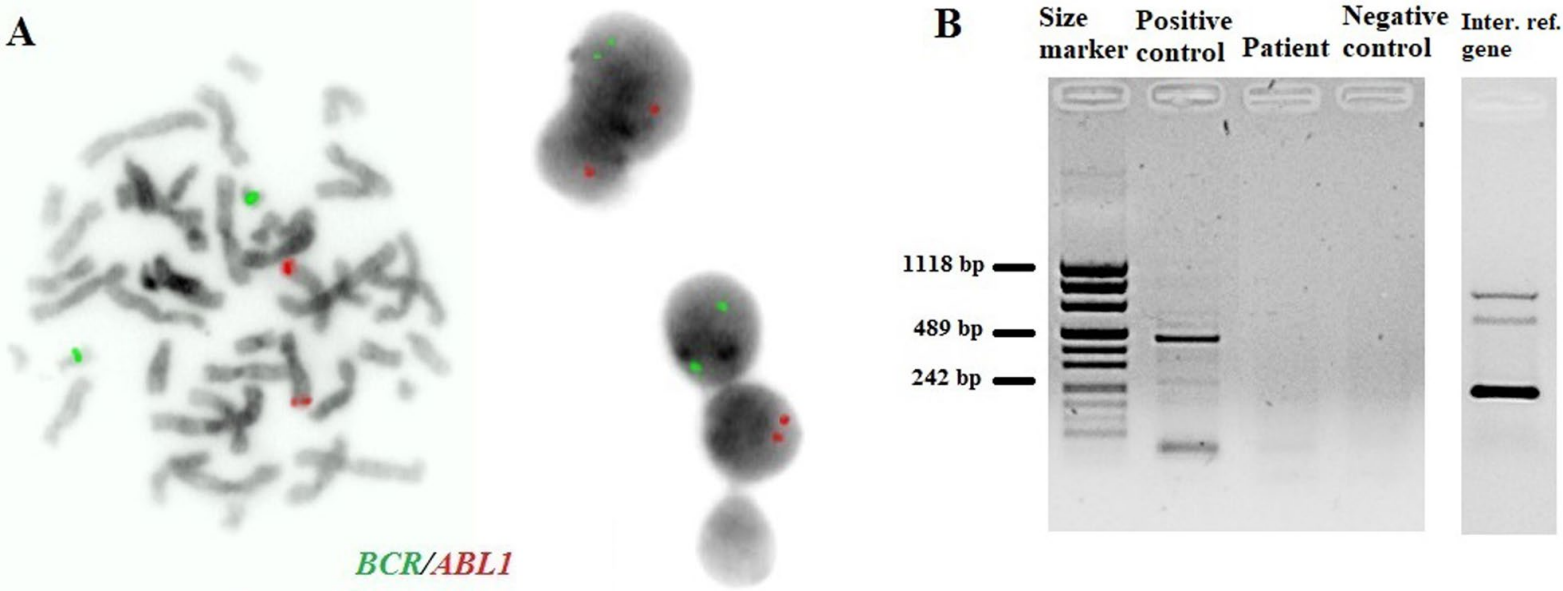
**A**—FISH study on day 33 of treatment, **B**—RT-PCR test on day 33 of treatment. Lane 1: size marker; lane 2: positive control; lane 3: patient; lane 4: negative control, lane 5: internal reference gene—ABL1

The patient’s induction therapy started according to the protocol IA-Pred. On the 8th day of treatment, the patient had a poor response to prednisone. Due to the presence of the *BCR /ABL1* fusion gene, further treatment was performed according to the EsPhALL 2009 protocol (European intergroup study of post-induction treatment of Philadelphia-chromosome-positive ALL). Imatinib at a dose of 300 mg/m^2^ daily was started on day 15 of treatment, but on day 28 was withheld due to hepatotoxicity (WHO grade III). Evolution of peripheral blood cell counts during therapy is presented in Table 1. In accordance to protocol, the patient's bone marrow was collected on days 15 and 33 of treatment. Examination of the bone marrow sample on day 15 revealed 15.4% blast cells in bone marrow morphology. Flow cytometry (FCM) revealed 23.48% of blasts. On day 33 (TP1), the bone marrow was already aplastic. Nevertheless, a PCR-MRD (Minimal Residual Disease) result was obtained. MRD in TP1 was low-positive (< 10^−4^). Bone marrow smear revealed a total of 2.6% of blasts. Despite the poor quality of the material in TP1, it was also possible to perform a FISH study (Fig. 3A) and RT-PCR test (Fig. 3B). Both molecular and cytogenetic tests were negative. According to the EsPhALL 2009 protocol the boy should have been classified as poor risk Ph(+) ALL group because of PPR (prednisone poor responder) on the 8th day, but due to complete remission on day 33 (LBL 1.2%, PC-MRD < 10^–4^) he was classified as good risk Ph(+) ALL group. From about day 32 of treatment, the patient reported abdominal pain, constipation, nausea and vomiting. Physical examinations showed hepatomegaly and lazy intestinal peristalsis. The symptoms were most likely caused by paralytic intestinal obstruction after chemotherapy. Additionally, the patient developed a fungal infection of the bladder.

**Table 1:** Evolution of peripheral blood cell counts during therapy

	Admission	8th day of treatment	15th day of treatment	33th day of treatment
White blood cells [μl]	206,900	8360	2750	660
Neutrophils [μl]	13,660	3430	1920	420
Lymphocytes [μl]	155,400	4080	710	220
Monocytes [μl]	3780	600	120	20
Eosinophils [μl]	180	10	0	0
Basophils [μl]	24,990	20	0	0
LUC (large unstained cells) [μl]	33,880	220	10	0
Red blood cells [μl]	3,460,000	2,620,000	2,520,000	2,860,000
Hemoglobin [g/dl]	10.2	7.4	7.0	9.0
Hematocrit [%]	28.7	22.1	21.7	26.4
MCV (mean corpuscular volume) [fl]	83.0	84.3	86.4	92.4
Platelets [μl]	142,000	53,000	58,000	40,000
Peripheral blasts [%]	64	15	0	0

Due to the general condition of the patient, consolidation treatment was delayed by 25 days. After this time, according to the EsPhALL 2009 protocol the IB protocol was started and Imatinib was resumed. Another PCR-MRD test was performed on day 17 of treatment (TP1b) of the IB protocol. PCR-MRD in TP1b was negative. Therefore the patient continued chemotherapy without qualification for HSCT (Hematopoietic Stem Cell Transplantation). The patient after consolidation therapy was in haematological remission of ALL. The patient remains without a transplant for 8 months after diagnosis.

## Discussion and conclusions

The very rare e8a2 transcript (about 8% from 1% of non-typical *BCR/ABL1* transcripts) has been reported mainly in cases of chronic myeloid leukemia (CML) [8–13, 18]. Two cases have been reported in adult ALL [14, 15]. The e8a2 *BCR/ABL1* transcript could be associated with worse prognosis than the e13a2 or the e14a2 transcripts in CML patients. However, there were cases of good response to treatment with imatinib with an achievement of a major molecular response [8, 10, 12]. CML cases with this transcript that have been reported so far, additionally had insertions from *ABL1* intron 1b or 1a, from *BCR* intron 8 or another gene such as *PRDM12*, *MAST2* [11, 16, 17]. Only one patient with CML and e8a2 *BCR/ABL1* transcript had no additional insertions and after treatment with imatinib achieved a complete cytogenetic response [12]. In adult acute lymphoblastic leukemia one case was reported with insertion of 2 nucleotides from *ABL1* intron 1a [14]. One adult ALL woman that had *RALGPS1* exon 8 inserted into the fusion, was treated with FLAG-Ida (fludarabine, cytarabine, granulocyte-colony stimulating factor [G-CSF], idarubicin) and dasatinib and after re-induction therapy achieved hematological, cytogenetic and molecular remission [15]. Unfortunately, the e8a2 variant in adult ALL patients is so rare, that its impact on outcome remains unknown. To the best of our knowledge, our patient is the first pediatric ALL case with e8a2 *BCR/ABL1* transcript. Our case sequencing analysis revealed e8a2 *BCR/ABL1* transcript without any insertion. Creation of the e8a2 transcript by the exact fusion of *BCR* exon e8 to *ABL1* exon a2 could encode an oncogenic protein, therefore our patient was qualified for treatment with the EsPhALL 2009 protocol. Our patient achieved complete remission after 33 days of treatment. Molecular and cytogenetic studies in TP1 did not reveal the presence of the *BCR/ABL1* transcript. The PCR-MRD test in TP1b was negative, the patient did not require hematopoietic stem cell transplantation.

The presence of the *BCR/ABL1* fusion gene is considered an unfavorable genetic abnormality and is associated with poor prognosis but survival has improved with the development of TKI. Our case shows that atypical transcripts of *BCR/ABL1* also occur in cases other than CML or adult ALL. RT-PCR and sequencing are appropriate methods for identifying these atypical transcripts. Using both conventional cytogenetics and molecular methods, we are able to detect many genetic changes occurring in leukemias. It is important to identify them accurately and use this information to monitor the patient’s treatments. The monitoring of the presence and quantity of the *BCR/ABL1* transcript using the RT-qPCR method is a gold standard in monitoring of Ph + patients with chronic myeloid leukemia. This method can also be used in monitoring of Ph + ALL patients to assess treatment efficiency. For proper patient monitoring it is important to evaluate the type of transcript at the time of diagnosis. Detection of a rare atypical transcript may affect the patient's treatment and may be associated with a worse prognosis.

## Data Availability

The Sanger Sequencing data generated in the study has been submitted to NCBI GenBank BankIt with the accession number OL672741; https://www.ncbi.nlm.nih.gov/nuccore/OL672741. Reference sequences used in this study are available in the following link: https://www.ncbi.nlm.nih.gov/nuccore/NM_004327.4; https://www.ncbi.nlm.nih.gov/nuccore/NM_005157.6. https://www.ncbi.nlm.nih.gov/nuccore/MF925339.1/.

## Abbreviations

OS: Overall survival
TKI: Tyrosine kinase inhibitors
BCP-ALL: B cell precursor Acute Lymphoblastic Leukemia
CNS: Central Nervous System
FISH: Fluorescence in situ hybridization
RT-PCR: Reverse transcription-polymerase chain reaction
HSCT: Hematopoietic Stem Cell Transplantation
CML: Chronic myeloid leukemia
G-CSF: Granulocyte-colony stimulating factor
